# Supplementary material for: Mutations within the cGMP-binding domain of CNGA1 causing autosomal recessive retinitis pigmentosa in human and animal model
Source: Cell Death Discov. 2022 Sep 17;8:387. doi: 10.1038/s41420-022-01185-0 (PMC9482621; doi:10.1038/s41420-022-01185-0)
Supplement: Supplementary file 1 — Supplementary Tables of the Manuscript [file 41420_2022_1185_MOESM1_ESM.docx]

**Supplementary Tables of the Manuscript**

## Supplementary Table 1 - Retinal Degeneration Panel (105 Genes)

| *ABCA4* | *BEST1* | *DHDDS* | *IMPG2* | *OTX2* | *RBP3* | *SAG* |
| --- | --- | --- | --- | --- | --- | --- |
| *ADAM9* | *C2ORF71* | *EYS* | *IQCB1* | *PDE6A* | *RD3* | *SEMA4A* |
| *AIPL1* | *CA4* | *FAM161A* | *KCNJ13* | *PDE6B* | *RDH12* | *SLC24A1* |
| *BBS1* | *CABP4* | *FSCN2* | *KCNV2* | *PDE6C* | *RDH5* | *SNRNP200* |
| *BBS10* | *CACNA2D4* | *GNAT1* | *KLHL7* | *PDE6G* | *RGR* | *SPATA7* |
| *TRIM32* | *CACNA1F* | *GNAT2* | *LCA5* | *PITPNM3* | *RHO* | *TOPORS* |
| *BBS12* | *CDHR1* | *GPR143* | *LRAT* | *PRCD* | *RIMS1* | *TRPM1* |
| *MKS1* | *CERKL* | *GPR179* | *LRIT3* | *PROM1* | *RLBP1* | *TTC8* |
| *CEP290* | *CLRN1* | *GRM6* | *MERTK* | *PRPF3* | *ROM1* | *TULP1* |
| *BBS2* | *CNGA1* | *GRK1* | *MKKS* | *PRPF31* | *RP1* | *UNC119* |
| *ARL6* | *CNGA3* | *GUCA1A* | *NMNAT1* | *PRPF6* | *RP2* | *USH2A* |
| *BBS4* | *CNGB1* | *GUCA1B* | *NR2E3* | *PRPF8* | *RP9* | *ZNF513* |
| *BBS5* | *CNGB3* | *GUCY2D* | *NRL* | *PRPH2* | *RPE65* | *EFEMP1* |
| *BBS7* | *CRB1* | *IDH3B* | *NYX* | *RAX2* | *RPGR* | *PITX2* |
| *BBS9* | *CRX* | *IMPDH1* | *OAT* | *RB1* | *RPGRIP1* | *FOXC1* |

## Supplementary Table 2 - Primers used in this study

| **Primer** | **Sequence (5’- 3’)** | **Ta**  (^0^C) | **Amplicon**  **size (**bp**)** |
| --- | --- | --- | --- |
| *CNGA1-Ex-10-F* | GTATATCGTCATCATTATCCACTG | 60 | 855 |
| *CNGA1-Ex-10-R* | TCTGGGTACTCAGTTAGAGC |  |  |
| *Cnga1-Ex-9-F* | TGAGAGAGAAGTCCTGAGATACC | 63 | 400 |
| *Cnga1-Ex-9-R* | TGAGGTCATCTTTGGAGAGGC |  |  |

**Supplementary Table 3 – *CNGA1* gene variant identified in this study (HGVS nomenclature and variant classification applying ACMG Criteria)**

| **Chromosome** | **Position (hg38)** | **Ref Seq** | **Alt Seq** | **Gene** | **RS ID** | **HGVS Nomenclature** |
| --- | --- | --- | --- | --- | --- | --- |
| chr4 | 47936957 | C | T | CNGA1 | [rs544588016](https://varsome.com/variant/hg38/rs544588016?&annotation-mode=germline) | [NM_001379270.1:c.1525G>A](https://varsome.com/variant/hg38/CNGA1(NM_001379270.1):c.1525G%3EA?&annotation-mode=germline)  (p.Gly509Arg) |
| **ACMG Criteria** | **Justification** | | | | | |
| PS3 Strong | Well established in vitro functional studies (Jin et al., 2016- PMID: 26802146). | | | | | |
| PP3 Supporting | Pathogenic computational verdict based on 12 pathogenic predictions from BayesDel_addAF (Score - 0.4308), CADD (Score -26.1), DEOGEN2 (Score -0.8299), EIGEN (Score -1.0079), FATHMM-MKL (Score -0.9805), LIST-S2 (Score -0.996), M-CAP (Score -0.7232), MVP (Score -0.9722), MutationAssessor (Score -4.1), MutationTaster (Score -1), PrimateAI (Score -0.9237) and SIFT (Score -0) vs no benign predictions. – Meta predictors – All Meta predictors predicted this variant as pathogenic -REVEL (Score- 0.93); MetaLR (Score-0.9481); MetaSVM (Score-1.1063); MetaRNN (Score- 0.9325). | | | | | |
| PM2 Moderate | Variant is present at extremely low frequency in the control population – gnomAD exomes - Allele frequency - 0.000008029 (2/249086) – No homozygotes – Present in only Asian population (East Asian – 1 in 17974; South Asian – 1 in 30588); gnomAD genomes (Allele frequency - 0.00001972 (3/152130)) {East Asians, 1 in 5194 alleles (0.0001925); European (Non Finnish, 1 in 68,040 (0.00001470); African/African American, 1 in 41,400 (0.00002415)} | | | | | |
| PP1 Supporting | We have found this variant to co-segregate in autosomal recessive pattern (homozgyous) among individuals with RP in our family. Furthermore, the same variant reported to co-segregate in a Chinese family as compound heterozygous mutation along with another variation. | | | | | |
| Classification | Pathogenic (PS3 Strong + PM2 Moderate + PP1 Supporting + PP3 Supporting) | | | | | |
