# Supplementary material for: Mutations within the cGMP-binding domain of CNGA1 causing autosomal recessive retinitis pigmentosa in human and animal model
Source: Cell Death Discov. 2022 Sep 17;8:387. doi: 10.1038/s41420-022-01185-0 (PMC9482621; doi:10.1038/s41420-022-01185-0)
Supplement: Supplementary file 2 — Supplementary Figure legends [file 41420_2022_1185_MOESM2_ESM.docx]

**Sup-Fig.1.** **Sequence chromatogram of DKRRP2 family members:** *CNGA1* exon 10, depicting the homozygous mutation (c.1525G>A; p.Gly509Arg) in the affected family members (A, E, F), whereas all other unaffected family members (B, C, D, G, H) were heterozygous for this variant. The sequence of a control subject is given in I. The sites of the mutation are marked by arrows.

**Sup-Fig.2.** ***Cnga1^Y509C/Y509C^* mice are lacking CNGA1 protein**: Western blot staining of *Cnga1^Y509C/Y509C^* mouse retina at PM1 and PM6 using CNGA1- and CNGB1-antibodies. Β-Actin was used as control. N = 3 biological and technical replicates.

**Sup-Fig.3. *Cnga1^Y509C/Y509C^* mice show compromised outer segment morphology.** (A-F) Representative confocal images showing expression of rhodopsin (green) in retinal cross sections of wild-type retina at PM1 (A), and *Cnga1^Y509C/Y509C^* mouse retinas (B-F) at PM1, PM3, PM6, PM9 and PM12, illustrating the outer segment morphology. Rhodopsin staining shows a gradual reduction of rhodopsin signal in the mutant retina, revealing the compromised morphology of rod outer segments in the *Cnga1^Y509C/Y509C^* retina already at PM1. Cell nuclei were stained with DAPI (blue). OS, outer segments; IS, inner segments; ONL, outer nuclear layer; OPL, outer plexiform layer; INL, inner nuclear layer.
