# Supplementary figures and images for: Mutations within the cGMP-binding domain of CNGA1 causing autosomal recessive retinitis pigmentosa in human and animal model
Source: Cell Death Discov. 2022 Sep 17;8:387. doi: 10.1038/s41420-022-01185-0 (PMC9482621; doi:10.1038/s41420-022-01185-0)

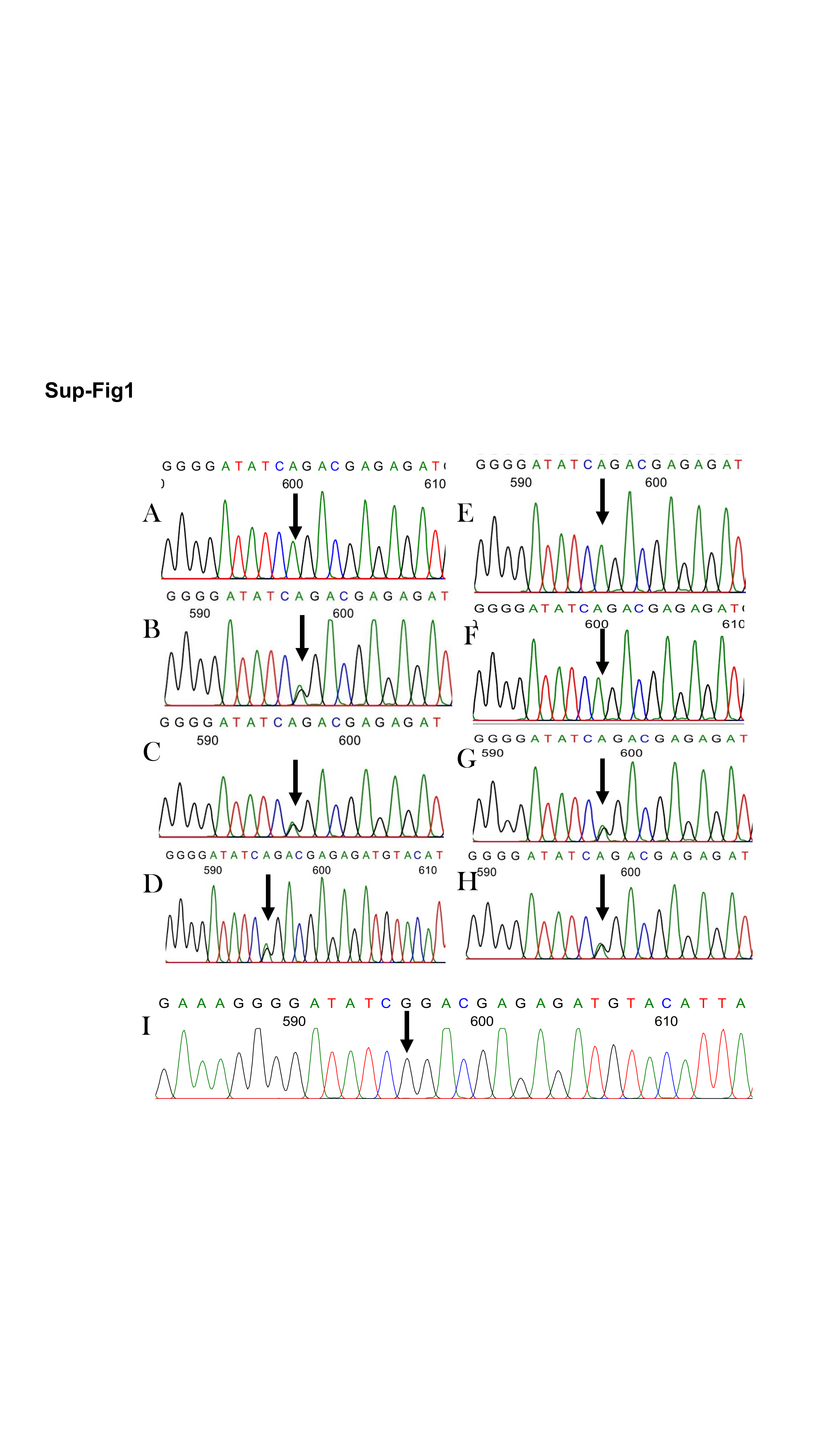

Supplement: Supplementary file 3 — Sequence chromatogram of DKRRP2 family members [file 41420_2022_1185_MOESM3_ESM.tif]

Sup-Fig2

WB

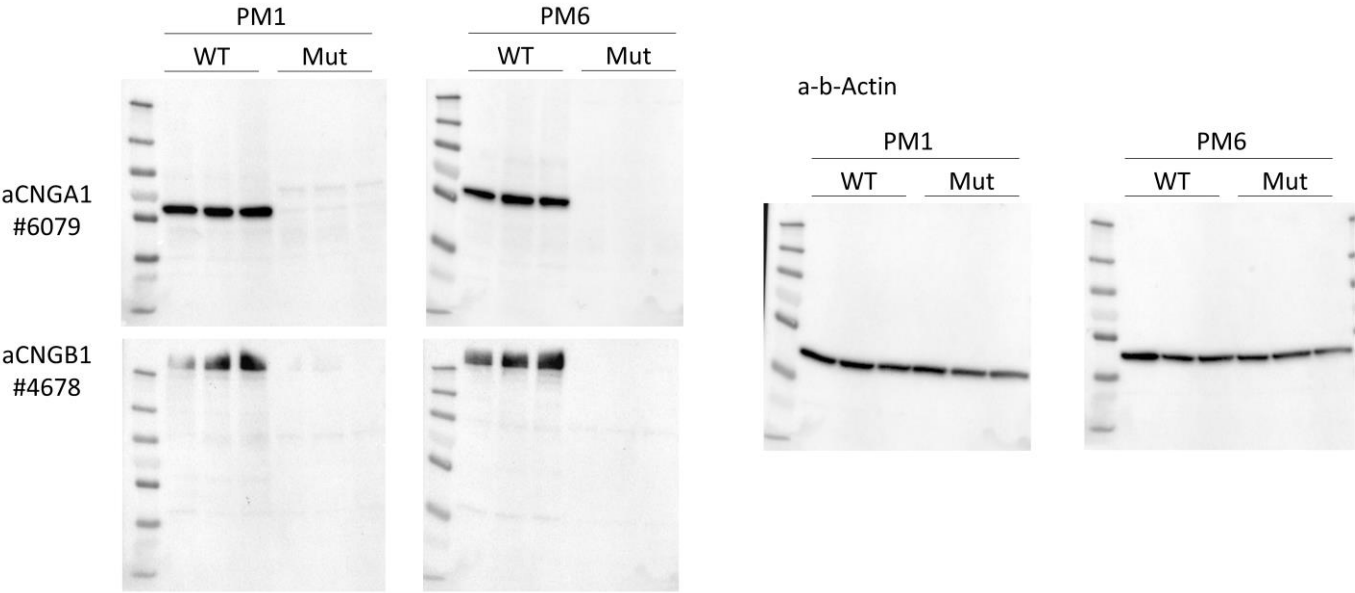

Supplement: Supplementary file 4 — WB originals [file 41420_2022_1185_MOESM4_ESM.pdf]

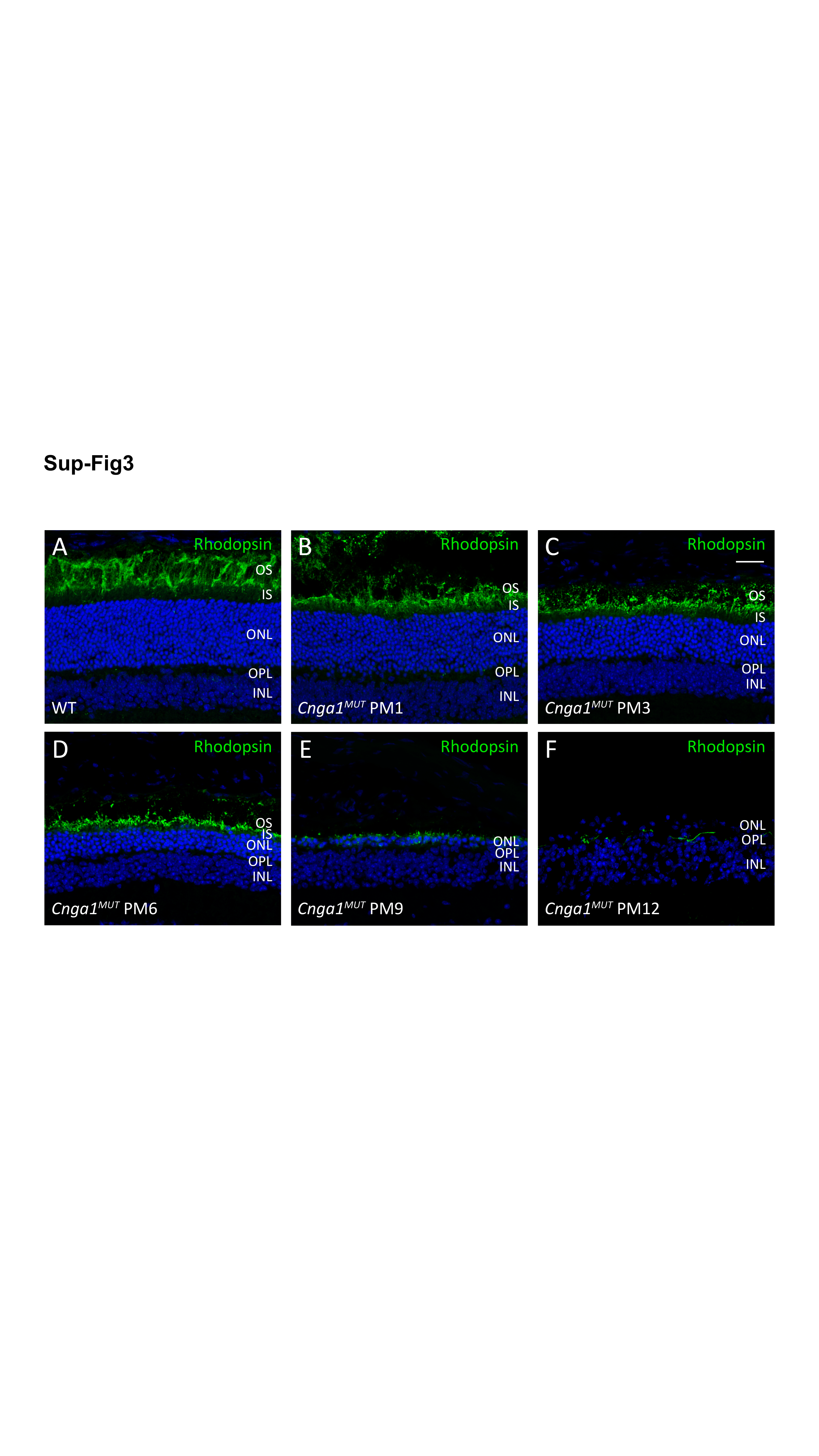

Supplement: Supplementary file 5 — Cnga1Y509C/Y509C mice show compromised outer segment morphology [file 41420_2022_1185_MOESM5_ESM.tif]
